# Investigation of a Brownfield Conflict Considering the Strength of Preferences

**DOI:** 10.3390/ijerph15020393

**Published:** 2018-02-24

**Authors:** Jing Yu, Ling-Ling Pei

**Affiliations:** 1 School of Economics and Management, Nanjing University of Science and Technology, Nanjing 210094, China; jingyu@njust.edu.cn; 2 Research Center for Low-Carbon Economy and Environmental Policy, Nanjing University of Science and Technology, Nanjing 210094, China; 3 Jiangsu Industrial Cluster Decision-Making and Consulting Research Base, Nanjing 210094, China; 4 Research Center for International Economy and Trade, Nanjing University of Science and Technology, Nanjing 210094, China; 5 School of Business Administration, Zhejiang University of Finance & Economics, Hangzhou 310018, China

**Keywords:** brownfield, conflict analysis, strength of preference, option prioritization

## Abstract

By employing the Graph Model for Conflict Resolution methodology, this paper models and analyzes a brownfield conflict that occurred at the Changzhou Foreign Language School in Jiangsu, China, in 2016. This conflict made national headlines when news reports revealed that a large number of students and staff suffered from health issues after the school moved to a new site that is built on recently restored land adjacent to the original “Chang Long Chemical” block. Since stakeholders in the conflict hold different strengths of preference, a new option prioritization technique is employed to elicit both crisp preferences and the strength of preferences for the decision-makers (DMs) in the conflict. The conflict analysis result is consistent with the actual trajectory of the conflict and provides strategic insights into the conflict. More specifically, equilibrium results suggest that the firm should have been required to thoroughly clean the site, the local government should not have relocated the school, and the environmental agency and other stakeholders should have closely monitored the firm’s activities. In short, strategic insights garnered from this case study indicate that positive interactions should be fostered among the local government, the enterprise, and the public to ensure sustainable brownfield land redevelopment in the future.

## 1. Introduction

What brownfield precisely entails differs from one jurisdiction to the next. Generally, brownfields refer to land previously used for industrial or commercial purposes with known or suspected pollution including soil contamination due to hazardous waste. In many countries, the redevelopment of brownfields is placed at a high priority on their political agendas. Brownfield redevelopment projects, however, are often problematic. For instance, environmental pollution and public health crises have frequently occurred in the process of brownfield land redevelopment, leading to numerous conflicts among different stakeholders in brownfield redevelopment projects. Therefore, brownfield redevelopment has attracted attention from governments, communities, environmentalist, scientists, and researchers around the world. Recently, brownfield-related conflicts have been extensively studied by scholars. For instance, Hipel et al. [1] proposed an innovative negotiation methodology for strategic and tactical decision-making in resolving brownfield redevelopment conflicts. Bashar et al. [2] investigated a brownfield property acquisition conflict using the fuzzy preference framework of the Graph Model. Blokhuis et al. [3] combined the conjoint analysis and game theory methods to model and analyze the underlying interaction structures in brownfield redevelopment projects. Walker et al. [4] applied the graph model for conflict resolution (GMCR) to analyze negotiations over the sale of a brownfield property with and without accounting for attitudes. Zhu et al. [5] developed an evaluation index system for brownfield redevelopment projects. Wang et al. [6] discussed a potential negotiation support system implementing numerical methods in the context of negotiating brownfield redevelopment projects. Kuang et al. [7] established a model of a brownfield redevelopment conflict employing the gray-based graph model for conflict resolution.

Among the aforementioned studies of brownfield related conflicts, a most frequently used methodology is the GMCR. The GMCR, as a flexible methodology for systematically modeling and analyzing conflicts, was originally put forward by Kilgour et al. [8] and Fang et al. [9]. One of the important advantages of GMCR is that it needs only decision-makers’ (DMs’) relative preference information, making it easy to calibrate a graph model and ideal in tackling strategic conflict with limited information. As such, the GMCR has been extended from different directions and employed to study various conflicts including brownfield disputes. The application of the GMCR to a real-world conflict generally consists of two steps: firstly, modeling the conflict within a formal (mathematical) framework; then, conducting stability analysis to predict possible equilibria of the conflict as well as other extended analysis methods such as coalition analysis [10,11] if appropriate. DMs’ relative preference is one of the key factors in both the modeling and analyzing processes. Since DMs may hold stronger preferences over some states in practice, based on crisp preferences [8,9], Hamouda et al. [12,13] developed the strength of preference (or three levels of preferences) under the framework of the GMCR. The efficient crisp preference eliciting method, option prioritization [14], was extended by Hou et al. [15] to model both crisp and three levels of preferences of DMs.

The main purpose of this paper is to capitalize on recent theoretic developments in the GMCR and establish a strategic conflict model for a brownfield dispute in China. This conflict occurred at the Changzhou Foreign Language School in 2016 shortly after it moved to a new site built on a recently restored brownfield and posed significant public health threat to students and staff in the school. This conflict was studied by Yin et al. [16] with a simple graph model. However, to better understand the interactions among the three stakeholders and capture their varying strength levels of preference, an expanded view is adopted to build an improved graph model for this dispute. A new option prioritization technique [14] is employed to elicit the three DMs’ preferences and calibrate the model. The analysis in this paper reveals more structural insights into the conflict and identifies viable options for the stakeholders to resolve the conflict.

The rest of the paper is organized as follows: In Section 2, the strength of preference framework of the GMCR and the strength option prioritization method are introduced. In Section 3, a real-world brownfield conflict is modeled and analyzed by employing the aforementioned methodologies. The paper concludes with some remarks in Section 4.

## 2. The GMCR with Strength of Preferences

In general, the crisp preference framework of GMCR is composed of a set of DMs N={1, 2, …, i, …, n−1, n}, a set of feasible states $S=\left\{s_{1},\;s_{2},\cdots ,s_{k},\cdots ,s_{t},\cdots ,s_{w}\right\}$, a set of oriented arcs $A_{i}\subseteq S\times S$, and a set of crisp preferences ${\left\{{≻}_{i},\;\sim_{i}\right\}}_{i\in N}$ on S for each DM, where $s_{k}{≻}_{i}s_{t}$ means that DM i prefers state $s_{k}$ to $s_{t}$, and $s_{k}\;\sim_{i}\;s_{t}$ indicates that DM i is neutral over the two states. Together, it can be described as $G=\langle N,\;S,\;{\left\{A_{i}\right\}}_{i\in N},\;{\left\{{≻}_{i},\;\sim_{i}\right\}}_{i\in N}\rangle$.

### 2.1. Strength of Preference and Stability Definitions

A strength of preference framework of GMCR is generally represented by $G=\langle N,\;S,\;{\left\{A_{i}\right\}}_{i\in N},\;{\left\{\gg_{i},{>}_{i},\sim_{i}\right\}}_{i\in N}\rangle$, where the triple of binary relations $\left\{\gg_{i},{>}_{i},\sim_{i}\right\}$ indicates DM i’s strength of preference, with the explanations that $s_{k}\gg_{i}s_{t}$ means that DM i strongly prefers state $s_{k}$ to $s_{t}$, while $s_{k}{>}_{i}s_{t}$ means that DM i mildly prefers state $s_{k}$ to $s_{t}$. Note that $\gg_{i}$ and ${>}_{i}$ are asymmetric, and $\left\{\gg_{i},{>}_{i},\sim_{i}\right\}$ is complete, which means that exactly one of $s_{k}\gg_{i}s_{t}$, $s_{t}\gg_{i}s_{k}$, $s_{k}{>}_{i}s_{t}$, $s_{t}{>}_{i}s_{k}$, or $s_{k}\;\sim_{i}\;s_{t}$ is true when a DM holds strength of preference over two states. Actually, $s_{k}{≻}_{i}s_{t}$ indicates either $s_{k}\gg_{i}s_{t}$ or $s_{k}{>}_{i}s_{t}$, while $s_{k}\geq_{i}s_{t}$ indicates either $s_{k}{>}_{i}s_{t}$ or $s_{k}\;\sim_{i}\;s_{t}$.

If DM i’s preferences on S are described in the triple of binary relations $\left\{\gg_{i},{>}_{i},\sim_{i}\right\}$, then DM i’s preference information is definite. Note that strength of preferences can also be represented by a matrix. For example, matrix ${ℛ}_{i}^{s}$ shows DM i’s strength of preferences on $S=\left\{s_{1},\;s_{2},\;s_{3},s_{4}\right\}$, which is equivalent with ($s_{1}{>}_{i}s_{2})\gg_{i}\left(s_{3}\;\sim_{i}\;s_{4}\right)$. As an example, “≫” in the first row and fourth column of ${ℛ}_{i}^{s}$ means that state $s_{1}$ is strongly preferred to $s_{4}$ for DM i.

$$\begin{array}{ccc} {ℛ}_{i}^{s}= & \begin{array}{c} \;\\ \begin{array}{c} s_{1}\\ s_{2}\\ s_{3}\\ s_{4} \end{array} \end{array} & \begin{array}{c} \begin{array}{cccc} s_{1} & s_{2} & s_{3} & s_{4} \end{array}\\ \left(\begin{array}{cccc} \sim & > & \gg & \gg \\ < & \sim & \gg & \gg \\ \ll & \ll & \sim & \sim \\ \ll & \ll & \sim & \sim \end{array}\right) \end{array} \end{array}$$

For $s_{k},\;s_{t}\in S$, and i∈N, based on the strength of preference structure, the set of all feasible states S can be divided into five sets. Accordingly, DM i’s reachable list from state $s_{k}$, $R_{i}\left(s_{k}\right)$, can be divided into five subsets, see Table 1 for details.

Notation $R_{i}^{+,++}\left(s_{k}\right)=R_{i}^{+}\left(s_{k}\right)\cup^{\text{​}}R_{i}^{++}\left(s_{k}\right)$ depicts the weak improvements (W-Is) from state $s_{k}$ for DM i. Let H⊆N (H≠ϕ) denote a subset of all DMs. The subset H’s UM list from state $s_{k}$, denoted by $R_{H}\left(s_{k}\right)=R_{H}^{++}\left(s_{k}\right)\cup^{\text{​}}R_{H}^{+}\left(s_{k}\right)\cup^{\text{​}}R_{H}^{=}\left(s_{k}\right)\cup^{\text{​}}R_{H}^{-}\left(s_{k}\right)\cup^{\text{​}}R_{H}^{--}\left(s_{k}\right)$, is actually a legal sequence of UMs by each DM in H, where a legal sequence means that a DM cannot move in succession. Coalition H’s W-I list from state $s_{k}$, denoted by $R_{H}^{+,\;++}\left(s_{k}\right)=R_{H}^{+}\left(s_{k}\right)\cup^{\text{​}}R_{H}^{++}\left(s_{k}\right)$, is a legal sequence of W-Is (M-Is or S-Is) by each DM in H. Let $\omega_{H}\left(s_{k},\;s_{t}\right)$ and $\omega_{H}^{+,\;++}\left(s_{k},\;s_{t}\right)$ denote the set of all last DMs in legal sequences of UMs and W-Is from state $s_{k}$ to $s_{t}$, respectively. The definitions of UM list and W-I list of a coalition are given below.

Definition 1 (UM list of a coalition): For coalition H⊆N and state $s_{k}\in S$, the coalition H’s UM list from $s_{k}$ is regulated inductively as $R_{H}\left(s_{k}\right)$, which meets the following conditions: (1) if i∈H and $s_{t}\in R_{i}\left(s_{k}\right)$, then $s_{t}\in R_{H}\left(s_{k}\right)$ and $i\in \omega_{H}\left(s_{k},\;s_{t}\right)$; and (2) if i∈H, $s_{t}\in R_{H}\left(s_{k}\right)$, $s_{v}\in R_{i}\left(s_{t}\right)$, and $\omega_{H}^{+,\;++}\left(s_{k},\;s_{t}\right)\neq \left\{i\right\}$, then $s_{v}\in R_{H}\left(s_{k}\right)$ and $i\in \omega_{H}\left(s_{k},\;s_{v}\right)$.

Definition 2 (W-I list of a coalition): For coalition H⊆N and state $s_{k}\in S$, the coalition H’s W-I list from $s_{k}$ is regulated inductively as $R_{H}^{+,\;++}\left(s_{k}\right)$, which meets the following conditions: (1) if i∈H and $s_{t}\in R_{i}^{+,\;++}\left(s_{k}\right)$, then $s_{t}\in R_{H}^{+,\;++}\left(s_{k}\right)$ and $i\in \omega_{H}^{+,\;++}\left(s_{k},\;s_{t}\right)$; and (2) if i∈H, $s_{t}\in R_{H}^{+,\;++}\left(s_{k}\right)$, $s_{v}\in R_{i}^{+,\;++}\left(s_{t}\right)$, and $\omega_{H}^{+,\;++}\left(s_{k},\;s_{t}\right)\neq \left\{i\right\}$, then $s_{v}\in R_{H}^{+,\;++}\left(s_{k}\right)$ and $i\in \omega_{H}^{+,\;++}\left(s_{k},\;s_{v}\right)$.

For the strength of preference structure, if a state is stable, then it is either strongly stable or weakly stable based on sanctioning strength. Note that strong and weak stabilities include only GMR, SMR, and SEQ because Nash stability does not involve sanctions. Definitions of solution concepts [12,13] referring to stabilities, strong stabilities, and weak stabilities are given in Table 2.

### 2.2. Strength Option Prioritization

In the option prioritization method in [14], each DM i possesses an ordered list of preference statements $P_{i}=\left[\Omega_{1},\;\Omega_{2},\;\cdots ,\;\Omega_{j},\;\cdots ,\;\Omega_{l},\;\cdots ,\;\Omega_{q}\right]$, where the preference statements that are considered more important for DM i appear earlier in the list. Each preference statement, which is expressed in terms of options and logical connectives, takes a “True” (T) or “False” (F) truth-value, at each state. Denote $\Omega_{j}\left(s\right)$ as the truth-value of the preference statement $\Omega_{j}$ at state s, and let $\Psi_{j}\left(s\right)$ be the score to state s based upon preference statement $\Omega_{j}$. Define

(1) $$\Psi_{j}\left(s\right)=\{\begin{array}{ll} 2^{q-j}, & if\;\Omega_{j}\left(s\right)=T\\ 0, & otherwise \end{array}\;\mathrm{and}\;\Psi \left(s\right)=\sum_{j-1}^{q}\Psi_{j}\left(s\right)$$

Then, the states can be sorted based on their scores. Specifically, $s_{k}{≻}_{i}s_{t}$ iff $\Psi \left(s_{k}\right)>\Psi \left(s_{t}\right)$, and $s_{k}{∼}_{i}s_{t}$ iff $\Psi \left(s_{k}\right)=\Psi \left(s_{t}\right)$.

Hou et al. [15] extended the above-mentioned option prioritization method to make it convenient to calculate the strength of preferences by adding weights to the preference statements. Specifically, if a DM strongly prefers a statement $\Omega_{l}$, where 1≤l≤q, then the notation $\Omega_{l}^{+}$ is applied to express the DM’s strong preference over statement $\Omega_{l}$. The weight is firstly defined by $W_{j}=2^{q-j}$. Taking $\Omega_{l}^{+}$ into account, the weight is redefined as

(2) $$W_{j}^{*}=\{\begin{array}{ll} 2^{q-j}+2^{q}, & if\;1\leq j\leq l\\ 2^{q-j}, & if\;l<j\leq q \end{array}$$

Then the score Ψ(s) to state s based upon the weight $W_{j}^{*}$ is defined in Equation (3), which is utilized if a DM strongly prefers the statement $\Omega_{l}$, denoted by $\Omega_{l}^{+}$. Otherwise, Equation (1) is employed.

(3) $$\Psi_{j}\left(s\right)=\{\begin{array}{ll} W_{j}^{*}, & if\;\Omega_{j}\left(s\right)=T\\ 0, & otherwise \end{array}$$

If a DM strongly prefers more than one statement, for instance, a DM may strongly prefer the statements $\Omega_{l_{1}},\Omega_{l_{2}},\;\cdots ,\;\Omega_{l_{g}}$, where $1\leq l_{1}<l_{2}<\cdots <l_{g}\leq q$, then the weight $W_{j}^{**}$ is defined by Equation (4) in consideration of $\Omega_{l_{1}}^{+},\Omega_{l_{2}}^{+},\;\cdots ,\;\Omega_{l_{g}}^{+}$.

(4) $$W_{j}^{**}=\{\begin{array}{cc} 2^{q-j}+g\cdot 2^{q}, & if\;1\leq j\leq l_{1}\\ 2^{q-j}+\left(g-1\right)\cdot 2^{q}, & if\;l_{1}<j\leq l_{2}\\ 2^{q-j}+\left(g-2\right)\cdot 2^{q}, & if\;l_{2}<j\leq l_{3}\;\\ \vdots & \vdots \\ 2^{q-j}+2^{q}, & if\;l_{g-1}<j\leq l_{g}\\ 2^{q-j},\; & if\;l_{g}<j\leq q \end{array}$$

Accordingly, Equation (5) shows the score Ψ(s) to state s based upon the weight $W_{j}^{**}$.

(5) $$\Psi_{j}\left(s\right)=\{\begin{array}{ll} W_{j}^{**}, & if\;\Omega_{j}\left(s\right)=T\\ 0, & otherwise \end{array}$$

For $s_{k},\;s_{t}\in S$, assume that $\Psi \left(s_{k}\right)\geq \Psi \left(s_{t}\right)$, then

(6) $$\{\begin{array}{c} \begin{array}{c} \begin{array}{cc} s_{k}\;\gg_{i}s_{t}, & if\;\Psi \left(s_{k}\right)-\Psi \left(s_{t}\right)\geq 2^{q} \end{array}\\ \begin{array}{cc} s_{k}{>}_{i}s_{t}, & \;\;if\;0<\Psi \left(s_{k}\right)-\Psi \left(s_{t}\right)<2^{q} \end{array}\\ \begin{array}{cc} s_{k}\;\sim_{i}\;s_{t}, & \;\;\;if\;\Psi \left(s_{k}\right)=\Psi \left(s_{t}\right) \end{array} \end{array} \end{array}$$

This strength option prioritization technique is effective and convenient for modeling both crisp preferences and the strength of preferences and is easy to implement into a decision support system.

## 3. Application to a Brownfield Conflict

Employing the crisp preference framework of the GMCR, Yin et al. [16] investigated a brownfield conflict that occurred in China in relation to the health problems of teachers and students at the Changzhou Foreign Language School. In this paper, a more integrative view is taken to restructure the DMs and their options in this brownfield conflict. Given the observation that the DMs display different strengths of preference, the recently developed strength of preference framework of GMCR and the corresponding option prioritization technique are employed to capture this important feature of the conflict. This improved model and analysis significantly complement the initial research in Yin et al. [16] and furnish more insights into the interactions of DMs and the conflict evolution.

Here is a brief introduction of the conflict. In May 2009, the government of Changzhou Xinbei District relocated the “Chang Long Chemical” block chemical-industry seat in order to improve the air quality. In March 2014, the original “Chang Long Chemical” block chemical-industry seat started to formally carry out land restoration. In September 2015, the Changzhou Foreign Language School moved to a new campus that is close to the repaired block. In the end of 2015, nearly 500 students in the Changzhou Foreign Language School showed signs of physical discomfort. The suspected reason was that the school was adjacent to the original “Chang Long Chemical” industrial land and the restoration operation had not met specifications. 

This event caused great attention from Changzhou Government, the Environmental Protection Agency, the teachers and students in the school, and the local citizens. The Changzhou Government organized experts to investigate the event and implement an emergency plan and an adjustment scheme. The investigation results revealed that there were volatile organic pollutants in the soil because the Black Peony Company used the “cover on spot” restoration method, instead of the “completely closed” method, for the land reparation project. Meanwhile, as an emergency scheme, there was a proposal to temporarily relocate the school. The Black Peony Company was required to clean the soil by fully enclosing the site. The Changzhou Government decided not to relocate the school, but the Environmental Protection Agency, the teachers and students in the school, as well as the local citizens started to supervise the Black Peony Company in order to prevent a similar accident from happening again. One can find details about this event in Liu et al. [17].

This event could be regarded as a conflict with multiple DMs. The Black Peony Company wanted to maximize economic benefits. The Changzhou Government pursued the maximization of both the social and economic benefits under the condition of ensuring the physical health of her citizens. The objective of the Environmental Protection Agency, the teachers and students in the school, and the local citizens was to fundamentally solve the environmental pollution problem.

### 3.1. DMs and Options

In the above-mentioned conflict, since the goals of the Environmental Protection Agency, the teachers and students in the school, and the local citizens are the same, they are regarded as one DM, which is represented by EPASC (the Environmental Protection Agency, the School, and the local citizens). Therefore, three DMs are involved in the conflict: DM 1: the Environmental Protection Agency, the School, and the local citizens (EPASC); DM 2: the Black Peony Company (BPC); and DM 3: the Changzhou Government (CG). The options of the three DMs are given in Table 3.

### 3.2. Feasible States and Graph Model

Since states with no option or with more than one option selected by DM 2 are infeasible and should be emitted, 16 feasible states finally remain as shown in Table 4. In Table 4, “Y” and “N” indicate that an option is taken or not taken by the DM controlling it, respectively.

**Figure 1:**
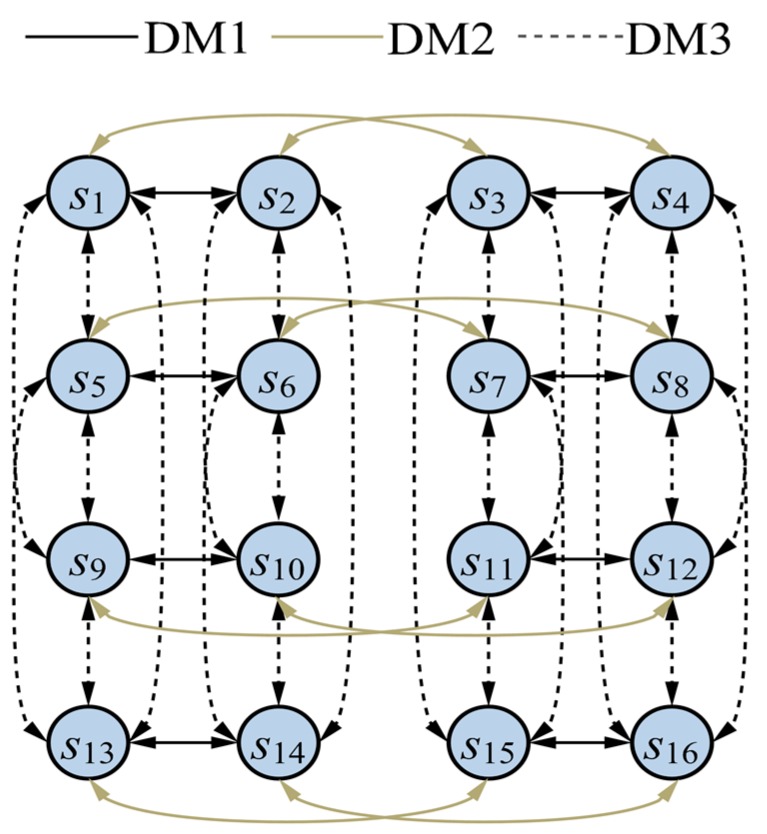
The integrated graph model.

Figure 1 shows the integrated graph model of the conflict. In Figure 1, the 16 circles indicate the 16 feasible states in Table 4, while the different directed arcs indicate the movements controlled by the corresponding DMs, where the arc tails represent the initial states, and the arrowheads represent the terminal states.

### 3.3. Strength of Preferences

Table 5 furnishes explanations of the prioritized preference statements in consideration of strength of preferences for each DM.

From Table 5, one can see that both DM 1 and DM 3 strongly prefer statement −B_1_, while DM 2 strongly prefers statement −C_2_. Employing Equations (2)–(6), all DMs’ strength of preference parameters can be calculated and ranked as shown in Table 6.

### 3.4. Stability Analysis

Based on all DMs’ strength of preferences rankings in Table 6, the stability analysis of the brownfield conflict can be calculated employing the stability definitions in Table 2. The stability analysis results are given in Table 7, where “√” indicates that, under a given general stability definition, the state in the particular row is stable for the corresponding DM or for all DMs, while “√^+^” indicates that, under a given strong stability definition, the state in a particular row is strongly stable for the corresponding DM or for all DMs. From Table 7, one can see that, when no strength of preference is considered, states $s_{2}$, $s_{3}$, $s_{4}$, $s_{6}$, $s_{7}$, $s_{8}$, $s_{11}$, $s_{15}$, and $s_{16}$ are GMR- and SMR-stable, and state $s_{12}$ is Nash-stable. After taking the DMs’ strength of preferences into account, states $s_{2}$, $s_{3}$, $s_{4}$, $s_{6}$, $s_{7}$, $s_{8}$, $s_{11}$, $s_{15}$, and $s_{16}$ are SGMR- and SSMR-stable, and state $s_{12}$ is still Nash-stable. This result indicates that state $s_{12}$ is more likely to be the equilibrium or resolution for the conflict, which is consistent with the reality. In reality, state $s_{12}$, where DM 1 (EPASC) chooses to supervise the process of land cleanup, DM 2 (BPC) employs the “completely closed” method, and DM 3 (CG) decides to punish DM 2 if DM 2 does nothing to improve the situation, turns out to be the final equilibrium and corresponds to the resolution for the conflict. Although states $s_{2}$, $s_{3}$, $s_{4}$, $s_{6}$, $s_{7}$, $s_{8}$, $s_{11}$, $s_{15}$, and $s_{16}$ are SGMR- and SSMR-stable after taking the DMs’ strength of preferences into account, these states are still less likely to be the equilibria or resolutions for the conflict since they are not strongly preferred by all DMs in the conflict. Take state $s_{2}$ as an example, although this state is strongly preferred by DM 2, this state is weakly preferred by both DM 1 and DM 3, so the conflict is not likely to settle at state $s_{2}$. From the above analysis, one can find that all DMs in the conflict can have an important influence on the evolution of the conflict, and a potential equilibrium or resolution for a conflict is generally a state that is beneficial for or favored by all DMs, other than by a particular DM or some DMs. One can also find that the strength of preferences of certain DMs may have little influence on the final stability analysis results.

### 3.5. Discussion

Public health crises in the process of brownfield land redevelopment have been frequent in the context of promoting industrial upgrading and de-industrialization in China. Reasons for this problem are ascribed to a lack of laws, standards, and policies for securing the process of brownfield land redevelopment. Take the toxic soil event in Changzhou, China, as a case study, Liu et al. [17] argue that the root cause of the problem is that, as pivotal stockholders of the brownfield land redevelopment project, local governments, enterprises, and property developers hesitate to pursue a prudent and secure land redevelopment process, which triggered a series of serious environmental issues and public health crises. The analysis in this paper is, to some degree, consistent with the discussions in [17]. If the Changzhou government and the Black Peony Company had communicated effectively to reach a consensus about carrying out the “completely closed” method to clean up the land, there would never have been any conflict. If the public and the Environmental Protection Agency know the brownfield reparation in advance, they would have started to supervise the project before any public health crisis occurred. Therefore, there should be a positive interaction between the local government, the enterprise, and the public to ensure sustainable brownfield land redevelopment in the future. The local government should formulate much stricter and securer plans for brownfield land redevelopment, monitor the whole process to make sure that developers follow the rules, and formulate and convey a penal code for the practitioners effectively. In addition, the local government’s policy making and the enterprise’s land cleanup project should be more open and transparent to the public. All the parties involved in the brownfield reparation project should interact positively, i.e., communicate efficiently, closely, and transparently, so as to make sustainable and secure decisions that are beneficial for all stakeholders.

Compared with [16], the modeling and analysis of the brownfield conflict in this paper make the following improvements:

(1) The teachers and students in the school as well as the local citizens are included as DMs. Since the Environmental Protection Agency, the teachers and students in the school, and the local citizens share similar goals, they are regarded as one DM which is represented by EPASC (the Environmental Protection Agency, the School, and the local citizens). In [16], the EPA rather than EPASC was modeled.
(2) DM 3 has three options (“Relocate”, “Don’t Relocate”, and “Punish”) in [16]; however, the options “Relocate” and “Don’t Relocate” are mutually exclusive. Therefore, in this paper, only the “Relocate” and “Punish” options of DM 3 are retained here.
(3) Twelve feasible states are identified in the conflict model in [16]. By further analyzing the actual progress of the conflict, this paper establishes a more appropriate model with 16 feasible states. Specifically, states $s_{11}$, $s_{12}$, $s_{15}$, and $s_{16}$ are added in this paper because DM 2’s Option B_2_ and DM 3’s Option C_2_ are not mutually exclusive, and these four states are feasible in reality.
(4) Although graph models for individual DMs were illustrated in [16], no integrated graph model for all DMs is shown. Figure 1 in this paper not only shows all possible actions of each DM but also clearly illustrates all possible evolution paths of the conflict.
(5) DMs’ strength of preferences, which are different from their crisp preferences, capture behavior and decision-making processes that are more consistent with reality. Moreover, the option prioritization method used to elicit DMs’ strength of preferences in this paper clearly shows why and how a DM’s preference demonstrates different levels of strength.
(6) The stability analysis results in Table 7 in this paper are more comprehensive and detailed, and it is thus easier for stakeholders to understand and garner more strategic insight into a given conflict. Specifically, the analysis in this paper significantly differs from that in [16]. In [16], states that are equivalent to states $s_{3}$, $s_{4}$, $s_{7}$, and $s_{8}$ in Table 4 satisfy GMR, SMR, and SEQ stability, and the state that is equivalent to state $s_{10}$ in Table 4 is Nash-stable. Based on this analysis, Yin et al. [16] argue that state $s_{4}$ in Table 4 is the equilibrium or resolution of the conflict. However, in this paper, the stability results show that states $s_{2}$, $s_{3}$, $s_{4}$, $s_{6}$, $s_{7}$, $s_{8}$, $s_{11}$, $s_{15}$, and $s_{16}$ are SGMR- and SSMR-stable, and state $s_{12}$ is Nash-stable. Thus, one can find that state $s_{12}$ is most likely to be the equilibrium or resolution for the conflict, which is also consistent with reality.

Therefore, the modeling and analysis of the brownfield conflict in this paper are more comprehensive and more consistent with what actually happened.

## 4. Conclusions

In the paper, the GMCR framework considering the strength of preferences is introduced and applied to model and analyze a brownfield conflict among three DMs. Moreover, the option prioritization methodology that can represent both crisp preferences and the strength of preferences is introduced and utilized to calculate DMs’ strength of preferences in the brownfield conflict model. The stability analysis results of the conflict show that state $s_{6}$, where EPASC (DM 1) decides to supervise the land restoration (i.e., select Option A_1_), BPC (DM 2) purifies the soil by fully enclosing the site (i.e., select Option B_2_), and CG (DM 3) decides not to move the school (i.e., not select Option C_1_) as well as not to punish BPC (i.e., not select Option C_2_), is consistent with the actual trajectory of the conflict, which demonstrates the feasibility and applicability of the conflict model. Moreover, one can gain a better and strategic understanding of the dispute by modeling and analyzing the conflict under the graph model and option prioritization methodologies. The study in this paper thus makes a significant contribution to brownfield studies and thus, more generally, environmental studies. The conflict analysis model established herein is an important supplement in the study of brownfield land redevelopment. Future research could be expanded to take uncertain preferences [18] or fuzzy preferences [19] into consideration to identify contradictions and to predict potential equilibria under circumstances of uncertainty so as to provide more strategic insights for both practitioners and researchers.

## Figures and Tables

**Table 1:** Subsets of S and subsets of $R_{i}\left(s_{k}\right)$.

Subsets of S	Description	Subsets of $R_{i}\left(s_{k}\right)$	Description
$\Phi_{i}^{++}\left(s_{k}\right)=\left\{s_{t}:\;s_{t}\;\gg_{i}s_{k}\right\}$	All states strongly preferred to state $s_{k}$ by DM i	$R_{i}^{++}\left(s_{k}\right)=R_{i}\left(s_{k}\right)\cap^{\text{​}}\Phi_{i}^{++}\left(s_{k}\right)$	All strong unilateral improvements (S-Is) from state $s_{k}$ for DM i
$\Phi_{i}^{+}\left(s_{k}\right)=\left\{s_{t}:\;s_{t}\;{>}_{i}s_{k}\right\}$	All states mildly preferred to state $s_{k}$ by DM i	$R_{i}^{+}\left(s_{k}\right)=R_{i}\left(s_{k}\right)\cap^{\text{​}}\Phi_{i}^{+}\left(s_{k}\right)$	All mild unilateral improvements (M-Is) from state $s_{k}$ for DM i
$\Phi_{i}^{=}\left(s_{k}\right)=\left\{s_{t}:\;s_{t}\;\sim_{i}\;s_{k}\right\}$	All states equally preferred to state $s_{k}$ by DM i	$R_{i}^{=}\left(s_{k}\right)=R_{i}\left(s_{k}\right)\cap^{\text{​}}\Phi_{i}^{=}\left(s_{k}\right)$	All equally unilateral improvements (E-Ms) from state $s_{k}$ for DM i
$\Phi_{i}^{-}\left(s_{k}\right)=\left\{s_{t}:\;s_{k}\;{>}_{i}s_{t}\right\}$	All states mildly less preferred to state $s_{k}$ by DM i	$R_{i}^{-}\left(s_{k}\right)=R_{i}\left(s_{k}\right)\cap^{\text{​}}\Phi_{i}^{-}\left(s_{k}\right)$	All mild unilateral disimprovements (M-Ds) from state $s_{k}$ for DM i
$\Phi_{i}^{--}\left(s_{k}\right)=\left\{s_{t}:\;s_{k}\;\gg_{i}s_{t}\right\}$	All states strongly less preferred to state $s_{k}$ by DM i	$R_{i}^{--}\left(s_{k}\right)=R_{i}\left(s_{k}\right)\cap^{\text{​}}\Phi_{i}^{--}\left(s_{k}\right)$	All strong unilateral disimprovements (S-Ds) from state $s_{k}$ for DM i

**Table 2:** Stability definitions in terms of strength of preferences.

**Stabilities**	**Definitions or Conditions**
Nash	$s_{k}\in S_{i}^{Nash}$, if and only if (iff) $R_{i}^{+,\;++}\left(s_{k}\right)=\phi$
GMR	$s_{k}\in S_{i}^{GMR}$, iff for each $s_{t}\in R_{i}^{+,\;++}\left(s_{k}\right)$, there is at least one $s_{v}\in R_{N-i}\left(s_{t}\right)$ to make $s_{v}\in \Phi_{i}^{--,\;-,\;=}\left(s_{k}\right)$
SMR	$s_{k}\in S_{i}^{SMR}$, iff for each $s_{t}\in R_{i}^{+,\;++}\left(s_{k}\right)$, there is at least one $s_{v}\in R_{N-i}\left(s_{t}\right)$ to make $s_{v}\in \Phi_{i}^{--,\;-,\;=}\left(s_{k}\right)$ and $s_{w}\in \Phi_{i}^{--,\;-,\;=}\left(s_{k}\right)$ for all $s_{w}\in R_{i}\left(s_{v}\right)$
SEQ	$s_{k}\in S_{i}^{SEQ}$, iff for each $s_{t}\in R_{i}^{+,\;++}\left(s_{k}\right)$, there is at least one $s_{v}\in R_{N-i}^{+,\;++}\left(s_{t}\right)$ to make $s_{v}\in \Phi_{i}^{--,\;-,\;=}\left(s_{k}\right)$
**Strong Stabilities**	**Definitions or Conditions**
SGMR	$s_{k}\in S_{i}^{SGMR}$, iff for each $s_{t}\in R_{i}^{+,\;++}\left(s_{k}\right)$, there is at least one $s_{v}\in R_{N-i}\left(s_{t}\right)$ to make $s_{v}\in \Phi_{i}^{--}\left(s_{k}\right)$
SSMR	$s_{k}\in S_{i}^{SSMR}$, iff for each $s_{t}\in R_{i}^{+,\;++}\left(s_{k}\right)$, there is at least one $s_{v}\in R_{N-i}\left(s_{t}\right)$ to make $s_{v}\in \Phi_{i}^{--}\left(s_{k}\right)$ and $s_{w}\in \Phi_{i}^{--}\left(s_{k}\right)$ for all $s_{w}\in R_{i}\left(s_{v}\right)$
SSEQ	$s_{k}\in S_{i}^{SSEQ}$, iff for each $s_{t}\in R_{i}^{+,\;++}\left(s_{k}\right)$, there is at least one $s_{v}\in R_{N-i}^{+,\;++}\left(s_{t}\right)$ to make $s_{v}\in \Phi_{i}^{--}\left(s_{k}\right)$
**Weak Stabilities**	**Definitions or Conditions**
WGMR	$s_{k}\in S_{i}^{WGMR}$, iff $s_{k}\in S_{i}^{GMR}$ and $s_{k}\notin S_{i}^{SGMR}$
WSMR	$s_{k}\in S_{i}^{WSMR}$, iff $s_{k}\in S_{i}^{SMR}$ and $s_{k}\notin S_{i}^{SSMR}$
WSEQ	$s_{k}\in S_{i}^{WSEQ}$, iff $s_{k}\in S_{i}^{SEQ}$ and $s_{k}\notin S_{i}^{SSEQ}$

**Table 3:** DMs and options.

DMs	Options
DM 1	A_1_: Supervise: Supervise the process of field repair
DM 2	B_1_: Retain: Maintain status quo, i.e., keep taking the “cover on spot” method
	B_2_: Improve: Improve the current situation by employing the “completely closed” method
DM 3	C_1_: Relocate: Relocate the school site temporarily
	C_2_: Punish: Punish DM 2 if DM 2 does nothing to improve the situation

**Table 4:** Feasible states.

DMs	Options	$s_{1}$	$s_{2}$	$s_{3}$	$s_{4}$	$s_{5}$	$s_{6}$	$s_{7}$	$s_{8}$	$s_{9}$	$s_{10}$	$s_{11}$	$s_{12}$	$s_{13}$	$s_{14}$	$s_{15}$	$s_{16}$
DM 1	A_1_	N	Y	N	Y	N	Y	N	Y	N	Y	N	Y	N	Y	N	Y
DM 2	B_1_	Y	Y	N	N	Y	Y	N	N	Y	Y	N	N	Y	Y	N	N
	B_2_	N	N	Y	Y	N	N	Y	Y	N	N	Y	Y	N	N	Y	Y
DM 3	C_1_	N	N	N	N	Y	Y	Y	Y	N	N	N	N	Y	Y	Y	Y
	C_2_	N	N	N	N	N	N	N	N	Y	Y	Y	Y	Y	Y	Y	Y

**Table 5:** DMs’ preference statements.

DMs	Statements	Descriptions
DM 1	(−B_1_)^+^	DM 1 strongly hopes that DM 2 does not select Option B_1_.
	(A_1_)^+^	DM 1 prefers Option A_1_.
	C_2_	DM 1 hopes that DM 3 selects Option C_2_.
	C_1_ & C_2_	DM 1 hopes that DM 3 selects both Options C_1_ and C_2_.
	C_1_	DM 1 hopes that DM 3 selects Option C_1_.
DM 2	(−C_2_)^+^	DM 2 strongly hopes that DM 3 does not select Option C_2_.
	B_1_	DM 2 prefers Option B_1_.
	−A_1_	DM 2 hopes that DM 1 does not select Option A_1_.
	C_1_	DM 2 hopes that DM 3 selects Option C_1_.
DM 3	(−B_1_)^+^	DM 3 strongly hopes that DM 2 does not select Option B_1_.
	A_1_	DM 3 hopes that DM 1 selects Option A_1_.
	C_2_	DM 3 prefers Option C_2_.
	−C_1_	DM 3 prefers to not select Option C_1_.

**Table 6:** DMs’ strength of preference rankings.

DMs	Preference Rankings
DM 1	$\left(s_{16}>s_{12}>s_{8}>s_{4})\gg (s_{15}>s_{11}>s_{7}>s_{3})\gg (s_{14}>s_{10}>s_{6}>s_{2})\gg (s_{13}>s_{9}>s_{5}>s_{1}\right)$
DM 2	$\left(s_{7}>s_{3}>s_{8}>s_{4}>s_{5}>s_{1}>s_{6}>s_{2})\gg (s_{15}>s_{11}>s_{16}>s_{12}>s_{13}>s_{9}>s_{14}>s_{10}\right)$
DM 3	$\left(s_{12}>s_{16}>s_{4}>s_{8}>s_{11}>s_{15}>s_{3}>s_{7})\gg (s_{10}>s_{14}>s_{2}>s_{6}>s_{9}>s_{13}>s_{1}>s_{5}\right)$

**Table 7:** Stability analysis results.

	Nash				GMR/SGMR				SMR/SSMR				SEQ/SSEQ			
	DM 1	DM 2	DM 3	E	DM 1	DM 2	DM 3	E	DM 1	DM 2	DM 3	E	DM 1	DM 2	DM 3	E
$s_{1}$						√^+^				√^+^				√^+^		
$s_{2}$	√				√	√^+^	√	√^+^	√	√^+^	√	√^+^	√	√^+^		
$s_{3}$		√			√^+^	√	√^+^	√^+^	√^+^	√	√^+^	√^+^		√		
$s_{4}$	√	√			√	√	√^+^	√^+^	√	√	√^+^	√^+^	√	√		
$s_{5}$						√^+^				√^+^				√^+^		
$s_{6}$	√				√	√^+^	√	√^+^	√	√^+^	√	√^+^	√	√^+^		
$s_{7}$		√			√^+^	√	√^+^	√^+^	√^+^	√	√^+^	√^+^		√		
$s_{8}$	√	√			√	√	√^+^	√^+^	√	√	√^+^	√^+^	√	√		
$s_{9}$			√				√				√				√	
$s_{10}$	√		√		√		√		√		√		√		√	
$s_{11}$		√	√		√^+^	√	√	√^+^	√^+^	√	√	√^+^		√	√	
$s_{12}$	√	√	√	√	√	√	√	√	√	√	√	√	√	√	√	√
$s_{13}$																
$s_{14}$	√				√		√		√		√		√			
$s_{15}$		√			√^+^	√	√^+^	√^+^	√^+^	√	√^+^	√^+^		√		
$s_{16}$	√	√			√	√	√^+^	√^+^	√	√	√^+^	√^+^	√	√		
